# Statin effects on immunoglobulin-G glycomic architecture and the link to cardiovascular disease

**DOI:** 10.21203/rs.3.rs-6112380/v1

**Published:** 2025-03-03

**Authors:** Azam Yazdani, Rosangela Hoshi, Mohammed Ammar, Chunying Li, Richard D. Cummings, Irena Akmačić-Trbojević, Ana Cindrić, Nina Šimunić-Briški, Ivan Gudelj, Robert Glynn, Paul Ridker, Daniel I. Chasman, Gordan Lauc, Olga Demler, Samia Mora

## Abstract

**Background:**

Immunoglobulin G (IgG) plays a critical role in immune defense yet our understanding of its role in cardiovascular disease (CVD) is evolving. Observational studies have correlated statin use with changes in IgG N-glycan structures. However, statin effects on IgG N-glycan changes have not been tested in randomized controlled trials, and their direct association with CVD remains unclear.

**Methods:**

IgG N-glycans were measured at baseline and after one year of randomized high-intensity statin interventions in 2 sub-studies of randomized trials: JUPITER (Justification for the Use of Statins in Prevention: an Intervention Trial Evaluating Rosuvastatin; NCT00239681; primary prevention; discovery, n = 239 participants); and TNT (Treating to New Targets; NCT00327691; secondary prevention; validation, n = 711). Using linear regression adjusted for baseline levels of IgG N-glycans and clinical risk factors (e.g., age, sex) as well as the occurrence of CVD during the year of follow-up, we investigated the one-year randomized effects of high-intensity rosuvastatin v. placebo on IgG N-glycans in JUPITER. Significant statin-IgG N-glycan associations were then validated in TNT with one-year randomized effects of high- v. low-intensity atorvastatin intervention. We examined the architecture of IgG N-glycan connectivity at baseline using a data-driven Bayesian network and compared it with the architecture after one year of randomized statin intervention. We then investigated whether the changes in IgG N-glycans triggered by statins were associated with incident CVD events.

**Results:**

We identified 5 IgG N-glycans (corresponding to core fucosylated, monosialylated, and disialylated IgG N-glycans) in JUPITER whose levels decreased significantly with statin versus placebo (false discovery rate < 0.05), with an approximate 11.3–25.9% reduction in the individual IgG N-glycan levels. Four out of the five IgG N-glycans altered by statin were validated in TNT. Furthermore, monosialylation and core fucosylation (glycan peaks, GP 16 and 18) were inversely associated with CVD in JUPITER (OR = 0.87 and 0.73 per standard deviation increase, 95% CI: (0.57, 0.98) and (0.55, 0.96) respectively), and validated in TNT. Despite the effect of statin therapy on certain IgG N-glycans, the overall architecture of the IgG N-glycan network remained unchanged after one year of statin intervention.

**Conclusion:**

High-intensity statin interventions decreased several specific IgG N-glycan levels without changing the overall architecture of IgG N-glycan connectivity. Two IgG N-glycans that were decreased by statins were inversely associated with CVD outcomes, suggesting that statins have effects on monosialylated and core fucosylated IgG N-glycans, which may affect their cardioprotective properties. These findings highlight a potential immunomodulatory role of statins through IgG N-glycan alterations that should be further investigated in relation to CVD.

## Introduction

Immunoglobulin G (IgG) plays a critical role in immune defense, acting as the most prevalent glycoprotein in human serum responsible for antibody-based immunity^1^. One of the essential modifications IgG undergoes is glycosylation, a process that adds carbohydrate chains, called IgG N-glycans, to its proteins. IgG N-glycosylation is the process of adding N-glycans to IgG. This post-translational modification, especially through attaching N-glycans, significantly influences IgG structure and function, particularly in its interactions with immune cells and receptors. IgG N-glycans represent important functional effectors^2^. IgG N-glycosylation affects the immunological function of IgG, including modulating pro- and anti-inflammatory signaling and cellular immune response^3^.

The relationship between IgG glycosylation and atherosclerotic cardiovascular disease (CVD) is of growing interest, especially as inflammation is a known driver of atherosclerosis^4^. Recent observational studies suggest that the use of statins, which are primarily used to lower low-density lipoprotein (LDL) cholesterol and reduce the risk of CVD, may also affect IgG N-glycan structures^5^. Due to the limitations of observational studies, it remains unclear whether statins directly alter IgG glycosylation and if these changes are linked to future cardiovascular outcomes. Further research, especially from randomized controlled trials, is essential to determine whether statins can modify CVD-associated IgG N-glycans and if these changes have clinical relevance.

Using data from two different randomized trials evaluating high-intensity statin therapy in primary and secondary prevention populations, we examined the effect of statins on IgG N-glycans at the individual IgG N-glycan level. Additionally, we investigated whether changes in IgG N-glycans were associated with incident CVD events. In addition to evaluating the effect of statins on individual IgG N-glycan levels, we assessed their impact on the IgG N-glycan network architecture. This approach provides a holistic understanding of how statins influence not only individual IgG N-glycans but also the broader system-level properties of IgG glycosylation. Even if statins alter individual IgG N-glycan levels, the overall architecture might remain stable, suggesting that the core biological functions of IgG are preserved. By examining the architectural level, we aimed to determine whether statins affect the systemic organization of IgG N-glycans.

## Methods

### Data Availability.

The data collected for this study was from 2 randomized controlled clinical trials. Requests to access the dataset from qualified researchers trained in human subject confidentiality protocols should be sent to the Steering Committees of the parent trials.

### Study Populations.

IgG N-glycans were evaluated at baseline and after one year of randomized high-intensity stain interventions in 2 sub-studies of randomized trials. The discovery population was a sub-study of the Justification for the Use of Statins in Prevention: an Intervention Trial Evaluating Rosuvastatin (JUPITER, NCT00239681) trial with 239 participants with IgG N-glycan measurements at baseline and year-1. JUPITER (URL: https://www.clinicaltrials.gov) was a randomized, double-blind, placebo-controlled trial that tested high-intensity 20 mg/day rosuvastatin versus placebo for primary CVD prevention (median follow-up 1.9, maximum 5 years) in participants with elevated high-sensitivity C-reactive protein (hs-CRP 2 mg/L or higher) and average to low levels of LDL cholesterol (<130 mg/dL). The validation cohort was a sub-study of the Treating to New Targets (TNT, NCT00327691) trial among 711 participants with IgG N-glycan measurements at baseline and year-1. TNT (URL: https://www.clinicaltrials.gov) was a randomized, double-blind, controlled trial comparing the efficacy of high-dose (80 mg/day) versus low-dose (10 mg/day) atorvastatin for the secondary prevention of CVD events (median follow-up 4.9 years), in patients with clinically evident coronary heart disease.

In both studies, participants provided written informed consent at the time of enrollment, and the study was approved by the local research ethics committee or institutional review board at each center and by the Mass General Brigham institutional review board (Boston, MA). The first and senior authors had full access to all data in the study and take responsibility for their integrity and data analysis.

The primary study design consisted of two nested CVD case-control studies with matched pairs based on age and sex from participants in two randomized statin trials. When assessing the effect of statins on IgG N-glycans, the case and control data were pooled and analyzed by randomized statin assignment. When examining the association between IgG N-glycans and CVD, the data was analyzed as a paired case-control study.

### IgG N-Glycan Measurements.

Plasma samples at baseline and one year from each study were placed in random order throughout 96-well plates. Additionally, for quality control (QC) and to avoid experimental biases, each plate contained 5 wells with duplicated samples from the same plate, 5 wells with duplicates from other plates, and 5 wells with aliquots of standard plasma sample (pooled plasma from healthy volunteers) to further control the repeatability of the procedure. Laboratory personnel were blinded to case or timepoint status.

IgG N-Glycan profiling and IgG N-glycan data preparation are reviewed in detail elsewhere^6,7^. Briefly, IgG was isolated from individual plasma samples using CIM r-Protein G LLD 0.2 mL Monolithic 96-well plate^8^, while IgG N-glycans were released by peptide: N-glycosidase F^6,9^. Prepared samples were sent to the processing laboratory where they were stored at −20°C until ultrahigh-performance liquid chromatography analysis was performed. All chromatograms were separated in the same manner into 24 distinct biantennary complex IgG N-glycans. The amount of IgG N-glycans at each peak was expressed as the percentage of the total integrated area. In addition to these 24 directly measured IgG N-glycan peaks, 8 IgG N-glycosylation traits were calculated by summing the relative areas of IgG N-glycans with shared structural features, representing the percentage of IgG N-glycans with those features in the total IgG N-glycome^7^: agalactosylation, monogalactosylation, digalactosylation, asialylation, monosialylation, disialylation, bisecting N-acetylglucosamine, and core fucosylation.

### CVD Outcomes.

In both studies, CVD outcomes were prospectively ascertained and confirmed through medical review by the respective clinical trial endpoint committees^10,11^.JUPITER cases were defined as incident myocardial infarction, stroke, coronary revascularization, unstable angina requiring hospitalization, or death. TNT cases were defined as nonfatal non–procedure-related myocardial infarction, resuscitation after cardiac arrest, fatal or nonfatal stroke, and coronary heart disease death.

### Clinical and Biomarker Risk Factors.

Baseline questionnaires were used to collect sex, age, ethnicity, use of non-randomized medications, hypertension, smoking, and other relevant aspects of health history. LDL cholesterol concentrations were calculated by the Friedewald equation when triglycerides were <400 mg/dL and measured by ultracentrifugation when ≥400 mg/dL^12–14^.

### Statistical Methods.

All analyses were performed using JUPITER as the discovery cohort, with a selection criterion of Benjamin-Hochman false discovery rate (FDR) < 0.05. The findings were validated in the validation cohort, TNT, using a p-value < 0.05. All individual IgG N-glycan peeks at baseline and year-1 were log-transformed, winsorized (to reduce the effect of possibly spurious outliers), and standardized to mean = 0 and scaled to standard deviation (SD) = 1 to allow for comparison of the effect estimates. The winsorization function automatically calculated the 5^th^ and 95^th^ percentiles and replaced values outside this range with the corresponding thresholds. This method limits the impact of outliers while preserving the overall distribution of the data.

### The effect of one year of randomized statin treatment versus control on individual levels of IgG N-glycans.

In the discovery cohort, JUPITER, we fitted a linear regression model of year-1 IgG N-glycans on high-intensity statin treatment versus placebo, adjusting for baseline IgG N-glycan levels, and covariates sex, age, race, batch/plate, non-randomized use of statin, and the occurrence of CVD during the year of follow-up. We selected significant effects based on an FDR threshold <0.05. We replicated the findings in the TNT cohort, fitting a linear regression of year-1 IgG N-glycans on high- versus low-intensity statin treatment adjusting for baseline IgG N-glycans and covariates sex, age, race, and the occurrence of CVD. We considered findings with a p-value < 0.05 as validated. We also included the interaction term sex and statins as well as the interaction term ethnicity and statins in the model to assess ethnicity and sex interactions with statins.

For comparison, we estimated the effect of using statin for a year on LDL cholesterol by fitting a linear regression model of year-1 LDL on statin treatment adjusting for baseline LDL levels and covariates sex, age, race, non-randomized use of statin, and the occurrence of CVD during follow-up.

### The architecture of IgG N-glycan connectivity.

We adjusted the levels of IgG N-glycans at baseline and year-1 for the effect of covariates (sex, age, race, CVD occurrence during follow-up, and plate) and identified a data-driven Bayesian network of baseline and year-1 IgG N-glycans each separately at level 0.001 to reveal the IgG N-glycan connectivity. For constructing the networks, we used an order-independent implementation of the conditional independence structure, learning PC-algorithm^15^. For the comparison of Bayesian networks, we examined node connectivity. This was applicable because we had only 24 nodes and the Bayesian network was undirected.

### Association of the IgG N-glycans altered by statin therapy with CVD events.

To evaluate if the IgG N-glycans that were altered by statins were also associated with CVD events, we fit conditional logistic regression models for matched case-control data. In the discovery cohort, JUPTER, CVD cases, and controls were matched for sex and age (±2years). In the validation cohort, TNT, CVD cases, and controls were matched on a disease risk score^12^ and statin randomization (low versus high statin dose).

We first fit a model adjusted for covariates age and race. In the second model, we also considered additional covariates: LDL cholesterol, HDL (high-density-lipoprotein) cholesterol, hypertension, and smoking. The IgG N-glycan CVD associations in the discovery cohort JUPITER (p-value <= 0.05) were then validated in TNT. We also conducted a joint analysis of IgG N-glycans with CVD. Based on the IgG N-glycan architecture revealed by Bayesian network analysis, we assessed the association between directly connected IgG N-glycans and CVD in a single model.

## Results

### Participant Characteristics.

The characteristics of participants in the two cohorts are provided in Table 1 including the number of participants who developed CVD during the follow-up period of the study. Within each cohort, cases and controls were generally well-balanced except for smoking status and hypertension in JUPITER.

### The effect of statins on individual IgG N-glycan levels.

We identified five IgG N-glycan peaks (23, 17, 18, 21, and 16) that were significantly altered (FDR < 0.05) after one year of high-intensity statin intervention compared with placebo in JUPITER. These peaks correspond to three types of glycosylation: GP16, GP17 and GP18 to monosialylation (IgG N-glycans with one sialic acid residue), GP21 and GP23 to disialylation (IgG N-glycans with two sialic acid residues), and GP16, GP18, and GP23 to core fucosylation (presence of a fucose residue on the core N-acetylglucosamine). Supplementary Figure S1 represents the comparison of baseline levels of IgG N-glycans in the placebo and statin groups in JUPITER. Statin-induced IgG N-glycan alterations were replicated in the TNT cohort (p-value < 0.05), except for IgG N-glycan peak 18. The linear regression analysis revealed that all five peaks exhibited negative beta coefficients, indicating a decrease in their levels (Table 2). We observed no evidence of sex or ethnicity differences in the effects of statins on the IgG N-glycans.

The effect size of statins on IgG N-glycans ranged from −0.30 to −0.12 corresponding to approximately 25.9% to 11.3% lower IgG N-glycan levels (on average) than those in the control group. By comparison, the effect size on LDL cholesterol lowering was −1.38 and −0.94 in JUPITER and TNT respectively, corresponding to 74.8% and 60.9% reduction respectively.

**Figure 1:**
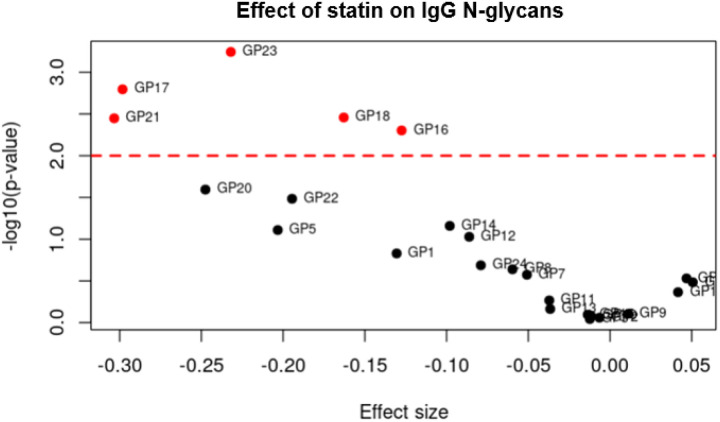
Comparison of effect size and significance levels in JUPITER. The effect size of high-intensity statin treatment versus placebo on IgG N-glycans, (*X*-axis) and the significance level, −log10(p-value), (*Y*-axis) in the JUPITER study. The red line represents the FDR threshold of 0.05 which accounts for multiple testing. The effect of the statin treatment on IgG N-glycan levels is mostly negative (i.e. reduced IgG N-glycan levels) for those that passed (red dots) or are close to the threshold level.

Statin had a negative effect on some individual IgG N-glycan levels, reducing them. This was true not only for those that passed the threshold but also for those near the statistical threshold (Figure 1).

**Figure 2:**
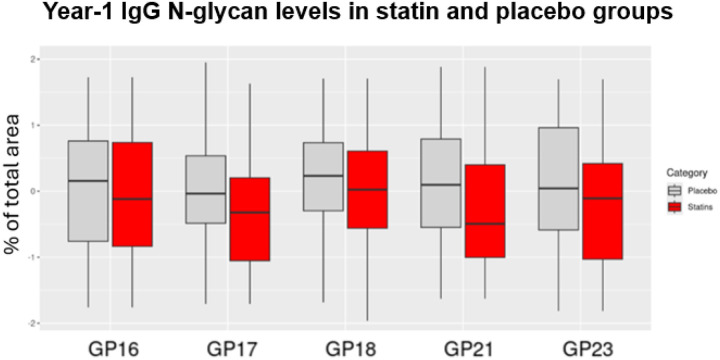
Comparison of year-1 levels of IgG N-glycan peaks significantly altered (FDR <0.05) by statin for the participants randomized to placebo or high-intensity statin in the discovery cohort, JUPITER, to illustrate the impact of statins on IgG N-glycan levels. GP stands for IgG N-glycan peak. The thick black lines in the boxes represent the median.

To show the reliability of the findings regarding the impact of high-intensity statin intervention on IgG N-glycan levels, we illustrated the specific effects of statin versus placebo on IgG N-glycan levels by comparing the year-1 levels of the five IgG N-glycans altered significantly by statin compared with placebo (Figure 2). This comparison in TNT is provided in (Supplementary Figure S2).

By considering the eight IgG N-glycosylation-derived traits defined from grouping IgG N-glycans with specific structural features^7^, we observed that IgG N-glycans associated with the traits of core fucosylation (GP 16, 18, 23), monosialylation (GP16, 17, 18), and disialylation (GP21, 23) were significantly altered by statins, whereas IgG N-glycans associated with other traits remained unchanged, (Supplementary Table S1).

### The effect of high-intensity statin intervention on IgG N-glycan architecture.

**Figure 3:**
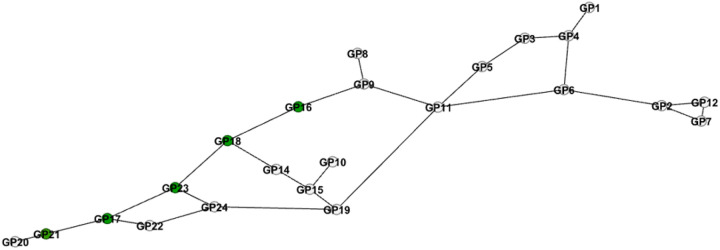
Baseline IgG N-glycan architecture. The data-driven Bayesian network of IgG N-glycans at level 0.001. Each node stands for an IgG N-glycan (GP), and each edge represents a partial correlation between the two corresponding GPs, which means the two GPs are directly connected after controlling for the effect of other GPs. If two nodes are not directly connected, it means the connectivity between the two corresponding IgG N-glycans can be explained by other IgG N-glycans in the study. The green GPs, which are in a path, are altered by statins. GP16 and 18 are the ones associated with CVD identified in the next section.

We identified IgG N-glycan architecture by using a data-driven Bayesian network. The architecture of IgG N-glycans at baseline is depicted in Figure 3. In the network, the nodes represent individual IgG N-glycans and the edges represent their connectivity identified by partial correlations, which are correlations corrected for the confounding effects of all other IgG N-glycans. Therefore, the edge between two nodes means that there is a direct connectivity between the two corresponding IgG N-glycans that cannot be attributed to other IgG N-glycans. We identified architecture at year-1 and compared it with the architecture at baseline based on node connectivity. We observed that the IgG N-glycan connectivity remained almost the same at the two time points, especially around the IgG N-glycans altered by statins.

After identification of the IgG N-glycan architecture, we mapped the IgG N-glycans whose levels were altered by randomized statins to assess their roles and determine whether they belonged to the same modules or were scattered across distinct regions. This analysis allowed us to evaluate whether statin-induced IgG N-glycan changes followed a coordinated pattern within specific functional clusters or reflected broader, unstructured alterations. We observed that the IgG N-glycans altered by statins were connected in a path, (Figure 3, the green nodes). Among these IgG N-glycans, only the disialylation IgG N-glycan peak 21 was located at the periphery, however, it had a high strength within the network, i.e., it is connected to the network with a low p-value^16^. For the strength of each connectivity in the network see (Supplementary Figure S3).

Most of the connectivity was validated in TNT, especially around IgG N-glycans whose levels were altered by statins (Supplementary Figure S4).

### Associations of the IgG N-glycans altered by statins with future CVD events.

By evaluating the association of incident CVD with the IgG N-glycan peaks altered by statins (listed in Table 2), we observed that monosialylated IgG N-glycan peaks 16 and 18, measured after one year of statin use, were negatively associated with CVD (p-value < 0.05) (Table 3). Both associations were replicated in TNT year-1, (p-value < 0.1).

Since IgG N-glycans 16 and 18 representing monosialylation and core fucosylation are directly connected, (Figure 3), we evaluated their associations with CVD at the same time (both in the model). We observed a reduction in the significance levels to 0.1 for both IgG N-glycans, (Supplementary Table S2).

## Discussion

This study is the first to demonstrate the randomized double-blinded controlled effect of high-intensity statins on IgG N-glycan levels and the potential relevance to CVD events, with a robust discovery-validation study design and utilizing two clinically distinct and contemporary populations (primary and secondary prevention) and two different high-intensity statin regimens. The present findings show the reproducible effects of statin on IgG N-glycosylation profiles and associations with CVD events. Our findings revealed that statins significantly decreased the levels of IgG N-glycans 16, 17, 18, 21, and 23. We also showed that the change in the levels of these IgG N-glycans does not change the overall IgG N-glycanarchitecture. Further, two of the IgG N-glycans (GP 16 and 18) that are related to monosialylation and core fucosylation were both reduced by statins and significantly associated inversely with incident CVD events. Whether these direct statin effects on IgG N-glycans may attenuate the statin cardiovascular benefit should be investigated in future studies.

The IgG N-glycans altered by statins are exclusively associated with the glycosylation traits of monosialylation, disialylation, and core fucosylation. Monosialylated IgG N-glycans are found on the fragment crystallizable region of IgG and are attached to the single conserved asparagine residue (Asn297) in the CH2 domain of the IgG heavy chain. Increased sialylation, represented by disialylated IgG N-glycans, is associated with anti-inflammatory properties. Moreover, altered sialylation patterns have been linked to changes in glycoprotein function, influencing inflammatory and immune pathways, further highlighting their potential role in CVD development and progression^17,18^. Core-fucosylated IgG N-glycans, which are also found on the same asparagine residue, affect antibody-dependent cellular cytotoxicity (ADCC), and their increase has been associated with anti-inflammatory properties. Fucosylation modulates inflammatory responses through altered interactions with FcγRIIIa and FcγRIIIb^3^. Disialylated IgG N-glycans promote an anti-inflammatory effect and play a key role in controlling immune responses and preventing excessive inflammation. A reduction in disialylated IgG N-glycans, associated with decreased galactosylation or sialylation, has been linked to increased inflammation. Core-fucosylated and monosialylated IgG N-glycans, which are typically less sialylated or not sialylated at all, contrast with disialylated IgG N-glycans, which are more sialylated and have increased anti-inflammatory effects by comparison^19,20^. These glycosylation traits are critical in modulating the anti-inflammatory and immunomodulatory properties of IgG. Therefore, the lowering effect of statins on these IgG N-glycans highlights the need for further investigation as well as studying the effects of other commonly used or novel cardiovascular preventive therapies.

One potential limitation of this study is that the primary design was two nested CVD case-control studies with matched pairs based on age and sex, hence this was a selected subset of the overall trial populations. To assess the effect of statins on IgG N-glycans, participants were grouped into statin and placebo groups. Since the original JUPITER trial randomly assigned participants to statins or placebo, this randomization likely mitigates potential biases in our study. Another limitation is the underrepresentation of ethnicities other than white, which may limit the generalizability of our findings. Future research on the effect of statins on IgG N-glycans in more diverse populations is warranted to enhance broader applicability. In addition, the IgG N-glycan changes were examined only over one year period. Despite the limitations, the strength of the study lies in the inclusion of two randomized trials with two different high-intensity statin regimens.

In summary, the current study demonstrated reproducible high-intensity statin effects on IgG N-glycosylation profiles (in particular, for the traits monosialylation, disialylation, and core fucosylation), without altering the overall IgG N-glycan architecture network. Further, two of the IgG N-glycans (related to monosialylation and core fucosylation) whose levels were reduced by statins were also associated inversely with incident CVD events. Whether these IgG N-glycan statin effects may attenuate the statin’s overall cardiovascular benefit is unknown. The IgG N-glycan alterations may be a secondary effect of statins rather than a causal factor in CVD outcomes. To fully understand the implications, further investigations are required, such as assessing the effect of statins over the longer term, as well as examining other commonly used or novel cardiovascular therapeutics. In addition, using the genetic information, we can identify the causal network of IgG N-glycans and extract more information about the architecture of IgG N-glycans, such as broadcasters, receivers, mediators, and confounders^21^. Furthermore, the link of causal networks to outcomes (here, CVD) facilitates the interpretation of the finding. Given the limited knowledge in the field of glycoimmunology and the widespread use of statins as cholesterol-lowering and cardiovascular preventive medications, their effects, which extend beyond lipid modulation to various immunological and inflammatory pathways, potentially influencing glycosylation processes, deserve additional investigation. Given the replicated inverse association of several traits with CVD events in primary and secondary prevention, this study opens the field of IgG glycomics as potential cardiovascular therapeutics that could be potentially developed for more targeted cardiovascular benefit.

## Figures and Tables

**Table 1.** Baseline characteristics of participants with IgG N-glycan measurements in the discovery and validation cohorts

Discovery cohort JUPITER	Overall (N = 239)	Placebo (N = 145)	High-intensity statin (N = 94)
Age, Mean (SD)	69.8 (8.4)	69.2 (8.4)	70.7 (8.3)
Women, N (%)	59 (24.6)	37 (25.5)	22 (23.4)
White, N (%)	209 (87.4)	123 (84.8)	86 (91.4)
Body Mass Index, kg/m^2	28.4 (6.7)	29.2 (7.5)	27.2 (4.9)
Hypertension, N (%)	135 (56.4)	87 (60)	48 (51.0)
Current smoker, N (%)	47 (19.6)	23 (15.8)	24 (25.5)
HDL cholesterol, mg/dL, Mean (SD)	51.6 (16.0)	51.3 (16.5)	52.0 (15.2)
LDL cholesterol, mg/dL, Mean (SD)	105.2 (18.1)	105.8 (16.7)	104.2 (20.1)
Incident CVD, N (%)	120 (50.2)	71 (48.9)	49 (52.1)
Validation cohort TNT	Overall (N = 711)	Low-intensity stain (N = 386)	High-intensity statin (N = 325)
Age, Mean (SD)	63.1 (8.3)	63.0 (8.6)	63.3 (7.8)
Women, N (%)	116 (16.3)	65.0 (16.8)	51 (15.6)
White, N (%)	652 (91.7)	350 (90.6)	302 (92.9)
Body Mass Index, kg/m^2	29.3 (5.1)	29.3 (4.8)	29.3 (5.4)
Hypertension, N (%)	488 (68.6)	267 (66.5)	221 (68)
Current smoker, N (%)	113 (15.8)	59 (15.2)	54 (16.6)
HDL cholesterol, mg/dL, Mean (SD)	45.5 (10.1)	44.7 (9.5)	46.5 (10.7)
LDL cholesterol, mg/dL, Mean (SD)	99.0 (17.4)	98.8 (17.3)	99.3 (17.6)
Incident CVD, N (%)	343 (48.2)	190 (49.2)	153. (7.1)

**JUPITER:** Justification for the Use of Statins in Prevention, an Intervention Trial Evaluating Rosuvastatin, **HDL:** high-density lipoprotein cholesterol, **LDL:** low-density lipoprotein cholesterol, **TNT:** Treating to New Targets

**Table 2.** IgG N-Glycan peaks (GP) that significantly changed over a 1-year period by randomized statin intervention in JUPITER and TNT

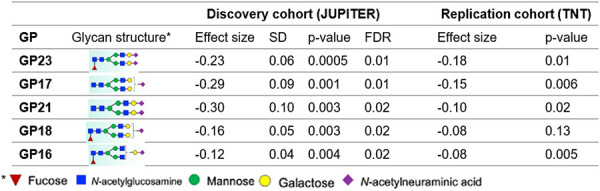

GPs are ordered first by FDR and then by effect size.

**Table 3.** Significant associations of IgG N-glycans altered by statin with incident CVD events

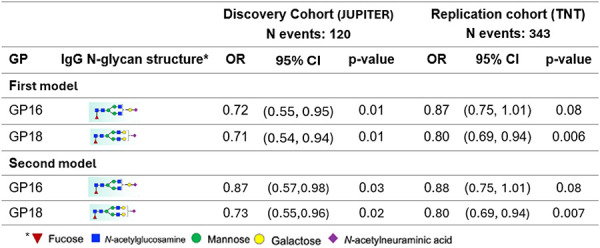

**OR:** Odds Ratio, **CI:** 95% Confidence Interval, **First model** is adjusted for sex, age, and race; **Second model** is adjusted for additional confounding factors LDL, HDL, hypertension, and smoking. The p-values of baseline GP16 and GP18 associations with CVD were 0.01 and 0.05 respectively. GP16 and GP18 are related to monosialylation as well as core fucosylation and were significantly associated with incident CVD. The other three IgG N-glycans that were altered by statin therapy (17,21, and 23) were not significantly associated with CVD events.
